# The Effect of Brain Breaks on Physical Activity Behaviour among Primary School Children: A Transtheoretical Perspective

**DOI:** 10.3390/ijerph16214283

**Published:** 2019-11-04

**Authors:** Hussein Rizal, Mawar Siti Hajar, Ayu Suzailiana Muhamad, Yee Cheng Kueh, Garry Kuan

**Affiliations:** 1 Exercise and Sports Science Programme, School of Health Sciences, Universiti Sains Malaysia, Kelantan 16150, Malaysia; husseinriz.usm@gmail.com (H.R.); mawar.usm@gmail.com (M.S.H.); ayu_suzailiana@usm.my (A.S.M.); 2 Unit of Biostatistics and Research Methodology, School of Medical Sciences, Universiti Sains Malaysia, Kelantan 16150, Malaysia; yckueh@usm.my; 3 Department of Life Sciences, Brunel University, London UB8 3PH, UK

**Keywords:** Brain Breaks^®^, exercise, intervention, cognitive process, behavioural process

## Abstract

Brain Breaks Physical Activity Solutions (BBPAS) is a web-based structured physical activity (PA) video that is specifically designed for school settings and can stimulate a student’s health and learning. The purpose of this study is to measure the effect of BBPAS on the stages of change, decisional balance, processes of change, self-efficacy and leisure-time exercise among Malay ethnic primary school children. A validated Malay version of three of the five constructs was derived with sound validity and was used in the present study. A total of 159 male and 163 female children aged 10 to 11 years old, mean (SD) = 10.53 (0.50), were recruited from two schools in Kelantan, Malaysia. Purposive sampling was used to divide the children into intervention (*n* = 177) and control (*n* = 145) groups. Children in the intervention group underwent BBPAS activity for an accumulated 30 min per week, while children in the control group were not involved in the BBPAS intervention. Mixed factorial analysis of variance (ANOVA) was used to examine the effect of BBPAS on the study variables. A mixed ANOVA showed significant changes (time effect) on cognitive process, *F*(1, 320) = 5.768, *p*-value = 0.017; behavioural process, *F*(1, 313) = 5.736, *p*-value = 0.017; and internal feeling, *F*(1, 312) = 6.050, *p*-value = 0.014. There was also a significant difference between groups on cons, *F*(1, 316) = 7.504, *p*-value = 0.007. A significant interaction effect was observed for stages of change, *F*(1, 319) = 7.861, *p*-value = 0.005; pros, *F*(1, 316) = 31.311, *p*-value = 0.001; internal feeling, *F*(1, 312) = 4.692, *p*-value = 0.031; and behavioural process, *F*(1, 313) = 7.312, *p*-value = 0.007. In conclusion, BBPAS was successful in improving four of the five constructs, and thus, should be recommended to be used in schools throughout Malaysia.

## 1. Introduction

Physical education contributes to the process of research in education as evidence of social and ethical personal development, and addressing the holistic education of children in physical, cognitive, emotional and social aspects [1]. This creates a need for educators to equip students with a holistic education that emphasises life skills like communication, cross-cultural collaboration and critical thinking [2]. The school environment is ideal for implementing physical activity interventions due to the possibility to reach a wide number of children who are spending most of their time in school. Therefore, a classroom activity break may provide a platform for students to undergo such a learning process as they age. Recently, some researchers have proven that physical activity in a school setting has helped to improve the students’ cognitive abilities and attitudes, and subsequently, their academic performance [3], which may be lacking in home environments. Therefore, an intervention is required to provide more involvement in physical activity among the young.

During the early years of implementation of the United Nation’s Sustainable Development Goals (SDGs), which were adopted in 2015, it sought to galvanise various international efforts to create faster progress towards achieving the SDGs’ bold aims [4]. Current projections show that many health-related SDG indicators on non-communicable diseases (NCDs), NCD-related risks and violence-related indicators will require a concerted shift away from what might have driven past gains-curative interventions in the case of NCDs. Instead, it should focus on multi-sectoral, prevention-oriented policy action and investments to achieve the aims of SDG #3, which is good health and well-being [4]. This led to the initiation of the Global Community Health (GCH) foundation, and in collaboration with HOPSports Inc., introduced the Brain Breaks Physical Activity Solutions (BBPAS) [5].

BBPAS is a web-based structured physical activity break that stimulates a student’s health and learning, as well as being specifically designed for the classroom setting to motivate students to enhance their theoretical lessons and provide an opportunity not only for them to be physically active during breaks, but to also learn new motor skills, language, art, music and different cultures [6]. The aim of the GCH foundation is to expand successful implementation of globally recognised, evidence-based health and wellness programs in communities while respecting local culture and customs [7]. They also select leaders who can educate and empower children to become premier agents of change, guiding them to create, share and practice health strategies that transform and improve the quality of life for others and themselves. The GCH foundation uses models from the Centres for Disease Control (CDC) and Whole School Whole Community Whole Child to utilise interventional strategies, expand projects and empower children respectively [7]. 

The transtheoretical model (TTM) is a psychological structure that ventures to describe the adoption and maintenance of healthy behaviours as a process that occurs over time [8]. The central aim of the TTM is to offer a clear understanding of the stages and processes of behavioural change [8]. It is an important issue for public health to understand why the majority of adults in industrialised countries are insufficiently active [9]. A recent meta-analysis has shown that the majority of studies support the hypothesis that the TTM can be applied towards the prevention of chronic diseases [10]. Exercise researchers have repeatedly recommended the TTM be applied to assess exercise behaviour in view of its applicability and generalisability [11,12]. 

Several psychological constructs of the TTM have been associated with exercise behaviour [13]. As the name suggests, the TTM model is comprised of different interconnecting models. The models are: (1) the stages of change, (2) processes of change, (3) decisional balance and (4) self-efficacy. The TTM suggests that changes in healthy behaviour occur in five stages, and that mechanisms of change incorporates cognitive and behavioural processes that individuals engage in at different stages [14]. In addition, individuals will weigh the pros and cons of engaging in physical activity and will generally experience increased self-efficacy as they advance through the stages of physical activity behavioural change [15]. Therefore, individuals may have higher levels of self-efficacy, have mentally outweigh the pros over the barriers of physical activity and incorporate more cognitive and behavioural thinking as they progress through the stages of change. It should be noted however, that the TTM is cyclical rather than linear; hence, relapses and regression in the stages are possible. 

In Malaysia, the TTM has been translated and validated to be used for the cessation of smoking [16,17]. The TTM application in Malaysia has also been studied for the reduction of body weight [18] and physical activity levels in women [19]. In addition, the TTM has also been translated and used in exercise behaviour among Malaysian secondary school students [20]. A transtheoretical framework may help in identifying the levels of the students’ motivation in wanting to participate in physical activity. Nevertheless, limited studies have been conducted using the transtheoretical model on the younger population [21]. Recently, translation and validation of the TTM on young ethnic Malay has been carried out [21] and is used in the present study. The use of the TTM as a theoretical framework can help the authors understand the process of the behavioural change of children through the BBPAS implementation. Therefore, the objective of the study is to measure the effect of BBPAS on the constructs of the TTM.

## 2. Materials and Methods 

### 2.1. Study Design, Recruitment and Sampling 

This study employed a quasi-experimental research design. Purposive sampling was used to recruit two high performance schools in the state of Kelantan, Malaysia. The two schools were assigned as the intervention (school A) and control (school B) groups. Students in the recruited classes were invited to participate. Students with prior injuries or conditions, such as heart problems, were excluded. Students who were currently engaged in long-term school activities that hindered consistent participation or had not acquired parental consent were also excluded. 

### 2.2. Participants

A total of 322 students (159 males and 163 females) aged 10–11 years old, mean (SD) = 10.53 (0.50), were recruited; 177 students from school A (intervention group; *n* = 177) and 145 students from school B (control group; *n* = 145). The selection of the students in the two schools were based on purposive sampling according to class. Equal selection of the students based on the class rank was carried out to avoid bias with regard to students’ academic performance. For example, the second-highest academically achieving class was chosen followed by the fourth, sixth and so on. Students with major examinations (year six) were omitted from the interventional study due to the restriction imposed by the Ministry of Education that forbids students with major exams to participate. Table 1 shows the demographics of the participants and baseline values for each analysis.

### 2.3. Procedures

This study gained approval from the Universiti Sains Malaysia (USM) Human Research Ethics Committee (USM/JEPeM/18020104). This study also conformed to the guidelines set by the International Declaration of Helsinki. Approval from the Ministry of Education and the State Education Department were also acquired. Upon volunteering, students and teachers from schools A and B were briefed about the objectives and procedures of the study. A validated questionnaire for the Malay children population was acquired based on a previous study using confirmatory factor analysis (CFA) to acquire sound validity and reported reliability in the form of Cronbach’s alpha and composite reliability [21]. A pre-test was conducted where the participants from both schools were asked to complete the questionnaires given. This similar process was repeated for the post-test. Students from school B (control group), on the other hand, were not involved in the BBPAS intervention. As the intervention group, students from school A underwent 12 weeks of BBPAS intervention from 27 June 2018 until 14 November 2018. The students were given the BBPAS intervention video for an accumulated time of 30 min per week. The intensity of the exercises typically ranges from low to moderate and include cultural to contemporary exercises from across the world, such as silat, Italian hip hop, wushu, Turkish Horon dance, taekwondo, aeroboxing, Greek Zorba dance and many more. The BBPAS video was projected on a screen using a projector in the school hall. Each week, a new BBPAS video was uploaded and shown to the students. BBPAS videos were readily available online for use (https://brain-breaks.com/). Teachers were present during the session, and if further medical assistance were required, students would be sent to the hospital by their teachers. Fortunately, no such cases occurred. 

### 2.4. Research Instruments

**Figure 1:**
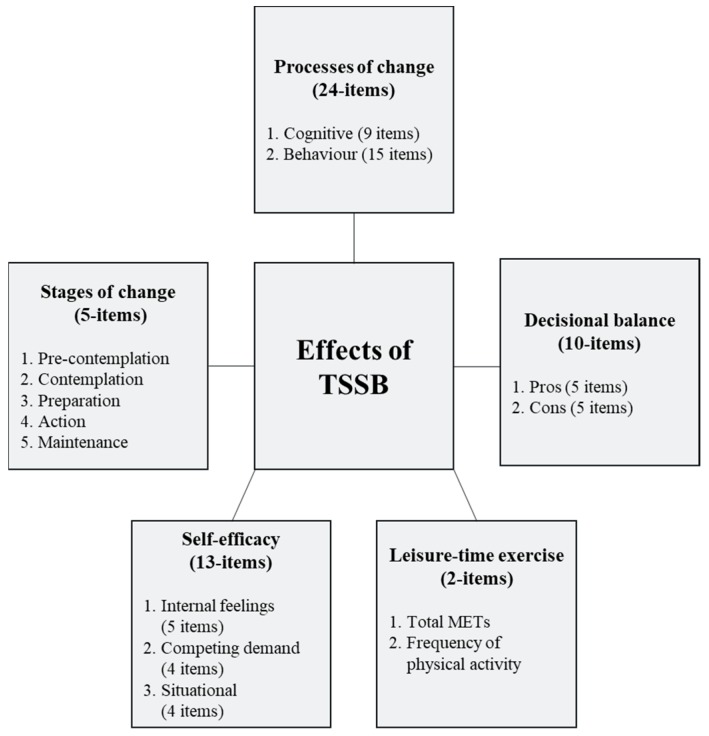
Conceptual framework of the validated Malay version of the transtheoretical model (TTM).

The research instruments used in this study are based on the TTM. The constructs include stages of change, processes of change, decisional balance and self-efficacy. In addition, Godin Leisure-Time Exercise Questionnaire (GLTEQ) was also included. Figure 1 demonstrates the conceptual framework of the TTM.

#### 2.4.1. Stages of Change

To assess physical activity behaviour, the stages of change questionnaire by Marcus et al. [12] was used. The five stages of the construct are: pre-contemplation, contemplation, preparation, action and maintenance [13]. In the dichotomous scale, students were asked to choose the statement that most closely applied to their activity level. Each corresponding statement reflected the stage of physical activity readiness in which the students were currently in. Stages of change scale was suitable to be used among school children as young as seven years old based on past literature [22,23].

#### 2.4.2. Processes of Change

The process of change questionnaire developed by Nigg et al. [24] was translated into Bahasa Melayu and validated for the present study. The original questionnaire had 30 items (10 factors). The internal consistency reliability of 0.60 to 0.90 for the two higher factors has been reported [24]. After validation, dramatic relief and environmental re-evaluation had low factor loadings and were removed. The Malay version of the processes of change scale has been validated using Malaysian primary school students with acceptable fit indices (comparative fit index; CFI = 0.939, Tucker Lewis index; TLI = 0.925, standardised root mean square residual; SRMR = 0.040, root mean square error of approximation; RMSEA = 0.030 (90% confidence interval; CI = 0.02–0.04), RMSEA *p*-value = 1.00) [21]. Each of the eight factors in the newly validated scale received a minimum of three statements that reflected its content and consisted of a five-point Likert scale ranging from (1) “not at all” to (5) “extremely”. The students were asked to tick the appropriate column to rate the degree of response. The validated revised Malay version of the processes of change scale used in the present study consisted of 24 items with eight factors [21].

#### 2.4.3. Decisional Balance

Plotnikoff et al.’s [25] 10-item decisional balance scale was translated into Bahasa Melayu and validated for the present study. This questionnaire consists of a five-point Likert scale ranging from (1) “not at all” to (5) “extremely”. The 10 items indicate how important each statement is with respect to the decision to be physically active or not to be physically active during a person’s leisure time. The decisional balance score was originally calculated by subtracting the average con score from the average pro score; the larger the score, the more pros the individual perceived compared to the cons [26]. However, in this study, two factors were derived, namely pros and cons, which were analysed separately. Both of these construct factors identify the positive and negative aspects of individuals’ behavioural change. The internal consistency reliabilities were reported as 0.82 for pros and 0.72 for cons [25]. The decisional balance scale has been validated using Malaysian primary school students and the CFA model showed a good fit based on the RMSEA and SRMR (CFI = 0.897, TLI = 0.864, SRMR (0.045) and RMSEA (0.038 (90% CI = 0.014–0.057), RMSEA *p*-value = 0.844) [21]. Although CFI and TLI indicated the model did not fit well to the data, the model for decisional balance was not subjected for further modification as the items were deemed suitable and important for the study population [21].

#### 2.4.4. Self-Efficacy

The three-factor, 18-item self-efficacy scale originated from Bandura [27] and was later revised for the Korean population [28,29]. The questionnaire was then translated into Bahasa Melayu and validated for the present study. This questionnaire consists of a five-point Likert scale that range from (1) “not confident at all” to (5) “completely confident” and originally had 18 items that correspond to a statement. Students were asked to rate in each of the blanks in the column regarding how certain they are that they can get themselves to perform their exercise routine regularly, i.e., three or more times per week. The original scale showed only one factor, with 77.5% of the variance explained by that factor [27]. The new scale developed for the Korean population was validated. The internal consistency for the scale was 0.91, while the two-week test–retest reliability was 0.86 [28]. The three factors were internal feeling, competing demand and situational. The validity of the Malay version of self-efficacy was confirmed based on CFA (CFI = 0.934, TLI = 0.915, SRMR = 0.042, RMSEA = 0.032 (90% CI = 0.011-0.047), RMSEA *p*-value = 0.977) [21]. Rizal et al. [21] reported that the validated revised Malay version of self-efficacy consisted of 13 items with three factors.

#### 2.4.5. Godin Leisure-Time Exercise Questionnaire (GLTEQ)

The GLTEQ by Godin and Shephard [30] was used to assess habitual weekly physical activity behaviour. The scale consists of three parts: strenuous exercise (heart beats rapidly), moderate exercise (not exhausting) and mild exercise (minimal effort). The weekly total leisure activity score (METs) was reported to obtain a more precise reading. METs is the objective measure of the ratio of the rate at which a person expends energy, relative to the mass of that person, while performing some specific physical activity compared to a reference, set by convention at 3.5 ml of oxygen per kilogram per minute, which is roughly equivalent to the energy expended when sitting quietly. The responses were then multiplied (in METs) by nine for strenuous, five for moderate and three for mild, and then totalled to acquire the total weekly leisure activity [30]. The reliability was reported as 0.94 for the strenuous activity score and the percentage of agreement was 74% for the total leisure time physical activity score using the kappa index [30]. In a recent study, the percentage of agreement between the test–retest classification was reported as 72%, and the kappa coefficient was reported as 0.40 with a 95% CI of 0.21–0.60 [31]. The scale has been widely used to measure the amount of physical activity among children [32]. It was deemed suitable to be used in the present study to measure the physical activity among primary school children.

### 2.5. Data Analysis

The statistical analysis was carried out using the Statistical Package for Social Sciences (SPSS) version 24.0 (IBM, Armonk, NY, USA). The numerical data are reported as means and standard deviations, and the categorical data as frequencies and percentages. Data were screened for missing values before the analysis. The final analysed sample involved 322 students. To measure the effects for each construct, a mixed factorial analysis of variance (ANOVA) at the two time points, i.e., at the beginning and end of the study period, were used. Time as the within-subject factor and group (intervention vs control) as the between-subject factor, as well as the interaction or main effect (time × group), were reported. The results were considered significant when *p* < 0.05. Baseline values for each measure were reported based on an independent *t*-test. 

## 3. Results

Table 2 shows the descriptive statistic of the stages of change across two time points, i.e., at the beginning and end of the study period. Significant findings related to the effects on groups, times and interaction (group × time) from a mixed factorial ANOVA are reported for the stages of change, pros, cons, cognitive process, behavioural process and internal feeling, as seen in Table 3. 

## 4. Discussion

### 4.1. The TTM as a Framework 

Previous studies had used the Attitudes toward Physical Activity Scale (APAS) [3,33,34,35] to measure children’s attitude and perceptions regarding various aspects of engagement in BBPAS [34]. These studies had shown positive findings in terms of promoting physical activity in school children [3,33,34,35]. The use of the APAS as a measuring tool focuses explicitly on the use of BBPAS as a physical activity intervention. 

In the present study, the TTM was used instead of APAS as the study’s framework. The framework measures the changes in behaviour throughout the intervention period based on processes of change, decisional balance and self-efficacy, with the stages of change being the underlying core that ties the rest of the constructs together. According to the TTM, there are studies for promoting exercise depending on individual stages in the process [36]. This is done to acquire a predictive model and algorithm that can create strategies to promote physical activity at targeted populations. 

Hence, correlations, discriminant function analysis and multivariate of variance (MANOVA) were often analysed in previous studies [15,28,37]. These studies would measure the difference between the constructs and produced a structure matrix to measure the relationship between physical activity behaviour (dependent) and the TTM (independent). However, in the present study, the changes seen in the TTM constructs (dependent) were individually assessed based on the BBPAS (independent) given at the two time points throughout the intervention period. 

Therefore, previous study parameters assessing the TTM differed from the present study whereby BBPAS was used as the intervention to evaluate its effect on the TTM. The present study adopted the protocols used by the recent BBPAS studies, such as Glapa et al. [34] and Popeska et al. [35], where the mixed ANOVA and repeated measures analyses of covariance (ANCOVA) were prioritised instead of MANOVA, as has widely been used in previous TTM studies, such as by Kim and Cardinal [14] and Han et al. [38]. In addition, baseline readings for each measure were all non-significant with the exception of stages of change. The control group saw a higher number of students in the later stages of change, especially in maintenance; however, they were reasonably balance for the other stages (refer to Table 2). Hence, no further adjustments to the sample were made.

### 4.2. Main Findings

The core of the TTM is the stages of change. In the present study, the majority of students from both groups were in the preparation stage and remained relatively consistent throughout the study in the intervention group, while the control group saw a small decrease. In the intervention group, pre-contemplators were drastically reduced, whereas, while the students in the action stage experienced a sharp increase, the maintenance stage showed a slight decrease. In the control group, students remained significantly stable over the same amount of time with only the pre-contemplators and contemplators increasing from the beginning to end stages. Consistent with past studies, BBPAS has been shown to promote physical activity [3,35], which in turn, led to an increase in the progression of stages. In addition, Nigg and Courneya [39] previously reported a significantly greater use of each process of change in 819 high school students classified in the higher stages of change compared to the lower stages of change. In the present study, the stages of change were significant for the interaction effect, which showed a positive effect due to administering BBPAS in the intervention group as opposed to no physical activity in the control group. Previous validation studies supported the claim that higher stages indicate higher physical activity participation [40]. 

The process of change consists of two higher factors, which are cognitive and behavioural processes. According to our knowledge, literature findings showed no study that used the TTM as a measuring tool for BBPAS as a physical activity intervention. Nonetheless, a previous study by Liu et al. [41] on Malay college students revealed the significant effects of physical activity on processes of change (*p* < 0.001). It can be clearly seen that the stages of change are the main factor contributing to the cognitive and behavioural processes of change that affect students’ involvement in physical activity [41]. Studies hypothesising theoretical constructs as mediators for physical activity, such as behavioural processes, cognitive processes, self-efficacy, decisional balance, social support and enjoyment, indicate that behavioural processes are the most likely mediators [40]. In the present study, both cognitive (time effect) and behavioural (time and interaction effects) processes were significantly increased in the intervention group. As the students’ progressed in the stages of change, there was also an increased use of cognitive and behavioural adaptions. 

The present study found no significant changes of BBPAS on the subscales of self-efficacy with the exception of internal feeling (time and interaction effects). Internal feeling includes items related to mental distress, such as anxiety and depression, which affects an individual’s confidence regarding physical activity performance. These findings conflicted with the work of Jeon et al. [42], who found that self-efficacy is a factor contributing to physical activity behaviour. Although the present study did not find a significant relationship between all the self-efficacy subscales and BBPAS, Cho [43] reported that individuals in the preparation and action stages had greater self-efficacy than individuals in the pre-contemplation and contemplation stages. In addition, Kim and Cardinal [44] reported that self-efficacy has a higher total effect on physical activity behaviour than decisional balance. This is in conflicting with this study, which showed that pros had significantly improved after they underwent BBPAS compared to self-efficacy. The difference in age may have resulted in varying results (as the present study was conducted on children), whereas most previous studies involved adults. Indeed, a recent review of reviews [45] described health status and self-efficacy as the “clearest correlates” of physical activity in both adults and adolescents [46]. Nonetheless, improving one’s self-efficacy in the early stages of change may help to increase physical activity levels no matter the age. 

For decisional balance, pros had a statistically significant interaction effect throughout the intervention period. Previous studies had indicated that pros and cons were key factors in regular exercise, and active students had greater positive factor scores for participating in exercise compared to students with low activity levels [47]. On the other hand, the cons of change were not significant for time and interaction effects; however, they were significant for the group effect. One explanation, which was also similar to the findings by Shaver et al. [48], is that the younger population may be less aware of the barriers when choosing to engage in activity, and would instead focus on the pros. Previous studies on disabled children showed that pros had positive association with physical activity, whereas experiencing barriers and the severity of the impairment were negatively associated [49]. Another study of Canadian adolescents found that self-efficacy and pros increased while cons decreased among children who were categorised in the higher stages of change [39]. Nonetheless, it was essential to form a positive decisional balance during the early stages of physical activity [50].

### 4.3. BBPAS as a Physical Activity Intervention

Education in schools may very well be a continuous process that is interchangeable between curricular education during classes and non-curricular education during BBPAS [3]. However, students spend more time on academic subjects instead of physical education [51]. Thus, a holistic approach towards education is needed whereby BBPAS enables general education continuity and the formation of confident, competent and responsible children for their own health [3].

The support of friends can also positively affect a student’s physical activity by providing them with a supportive social network that facilitates regular involvement in physical activity [44]. In the present study, BBPAS provided a platform for children to engage in physical activity together with friends, which may have improved enjoyment. Consistent with the findings by Uzunoz et al. [52], their research indicated that higher levels of physical activity self-efficacy and physical activity enjoyment correlated with higher levels of physical activity participation [46,53]. In addition, BBPAS had effectively helped to maintain the enjoyment of participating in physical activity over time, especially when it was performed together with peers as it helped to trigger a healthy competition [3]. However, in the present study, the total leisure activity score was not significant. This may be due to increased pressure from both groups to perform well academically as the intervention period took place during the second half of the year (June–November 2018). During this time, frequent exams may have hindered physical activity regardless of the BBPAS intervention. Although BBPAS has provided a platform for students to participate in physical activity, at other times, their level of activity remained relatively the same. Indeed, in a high-performance school, academic achievement takes priority over physical education. 

In addition, regular feedback would have provided valuable guidance to the process of change in these schools [54]. Examples of feedback from the researchers to each school are written summaries of the most important results of the interviews; overviews of the perceived barriers for the teachers and external pedagogical staff; and short, easily understandable animated videos of the most important results of the health and behavioural measures [55]. As such, rather than undergoing BBPAS in the given amount of time, as stipulated in previous studies, BBPAS was spread out over a duration of four months whereby lapses in the intervention may have negatively affected the results. With that said, the present study was based on a real-world study occurring in a real-world setting, which is considered the strength of this study. 

It should be noted that the main goal of BBPAS is to be used during classes for other subjects and only as a short active break [35]. This deferred from the present study as students conducted the session in the school hall due to the limitation of having only one projector. Most primary schools in Malaysia do not have a projector readily available in each classroom. This significantly inhibits the potential of BBPAS’s use, and it differs methodologically from past studies using BBPAS. In addition, teacher–student interaction in the use of BBPAS was also reduced in the present study as the researchers mostly initiated each BBPAS session accompanied by the presence of teachers. The effectiveness of the application of BBPAS is highly dependent on the teacher’s experience, creativity and personal motivation, as well as their skills regarding the use and implementation of information technology in their everyday teaching routine [56]. 

In the present study, BBPAS was considered a foreign project initiated by the researchers. Therefore, teacher and student cooperation may not have been equal to that of activities set by the school. In a study by Bartelink et al. [55], it aimed to not only evaluate, but also to support the process of change in schools. An intervention that revolves around on-going adaptions may continually lead to new needs, interests and opportunities in the school. In this context, Reiser et al. [57] stated that the implementation of a change is more successful and leads to greater ownership and commitment if it involves a process of mutual adaptation. In short, student and teacher cooperation are essential in the execution of the BBPAS intervention. Therefore, future studies should incorporate more teacher–student interaction of BBPAS to provide a more open platform. However, school policy (not allowing disturbances during classes) acted as one of the limitations of the present study.

BBPAS has been shown to improve physical activity behaviour for stages of change, pros (perceived benefits), cognitive process, behavioural process and internal feeling. Future studies should improve teacher–student interaction by allowing them to initiate BBPAS programs. However, regular guidance and feedback are required to maintain adherence. BBPAS is seen as a holistic approach for improving education by improving both cognitive and physical health in school settings.

### 4.4. Limitations

BBPAS is designed for the classroom setting; however, due to the limited number of researchers and helpers to conduct the sessions, BBPAS was conducted in the school hall. The big crowd during each session may have hindered the ability to ensure students’ correct movements throughout the sessions. Nevertheless, authors believe that this limitation had no significant impact on the findings of this study. The study was conducted in real-world situations, which the authors see as the strength of the study.

## 5. Conclusions

BBPAS has been shown to improve physical activity behaviour for stages of change, pros (perceived benefits), cognitive process, behavioural process and internal feeling. Future studies should improve teacher–student interaction by allowing them to initiate BBPAS programs since the teacher and student cooperation in this study may not have been equal to that of activities set by the school. Nonetheless, BBPAS is seen as a holistic approach to improve education by increasing both cognitive and physical health in school settings. 

## Figures and Tables

**Table 1:** Demographics of the participants (*n* = 322) and baseline reading for each measure.

Descriptive	Mean (SD)	Frequency (%)	Levene’s Test (*p*-Value)
Age (years)	10.53 (0.50)		
Gender: Male Female		159 (49.4) 163 (50.6)	
Groups: Intervention Control		177 (55.0) 145 (45.0)	
Construct:			
Stages of change			0.026
Pros			0.265
Cons			0.319
Cognitive			0.995
Behavioural			0.147
Internal feeling			0.138
Situational			0.770
Competitive			0.063
Total METs ^1^			0.351

^1^*Metabolic equivalents*.

**Table 2:** Frequency of the stages of change from the beginning to the end of the study period.

Stages of Change	Frequency (%)			
	Intervention		Control	
	Beginning	End	Beginning	End
Pre-contemplation	18 (10.2)	4 (2.3)	11 (7.6)	15 (10.3)
Contemplation	32 (18.1)	39 (22.0)	19 (13.1)	32 (22.1)
Preparation	81 (45.8)	81 (45.8)	61 (42.1)	52 (35.9)
Action	19 (10.7)	28 (15.8)	17 (11.7)	18 (12.4)
Maintenance	27 (15.3)	25 (14.1)	37 (25.5)	27 (18.6)

**Table 3:** Mixed factorial ANOVA and *F* statistics for each construct.

Construct	Group	Mean (SD)		Test Effect	*p*-Value	η^2^
		Pre-Test	Post-Test			
Stages of change	Int ^†^	3.03 (1.15)	3.18 (1.04)	Time Group Time × Group	0.388 0.305 0.005 *	0.002 0.003 0.024
	Con ^‡^	3.35 (1.21)	3.07 (1.23)			
Pros	Int ^†^	14.07 (3.81)	15.22 (3.51)	Time Group Time × Group	0.494 0.290 0.001 *	0.001 0.004 0.090
	Con ^‡^	15.79 (4.25)	14.32 (4.61)			
Cons	Int ^†^	11.66 (3.93)	11.87 (3.56)	Time Group Time × Group	0.070 0.007 * 0.304	0.010 0.023 0.003
	Con ^‡^	12.40 (4.16)	13.16 (4.64)			
Cognitive process	Int ^†^	24.52 (5.66)	24.23 (5.67)	Time Group Time × Group	0.017 * 0.701 0.103	0.018 0.001 0.008
	Con ^‡^	25.36 (5.77)	23.81 (6.77)			
Behavioural process	Int ^†^	36.56 (10.21)	39.86 (9.23)	Time Group Time × Group	0.017 * 0.559 0.007 *	0.018 0.001 0.023
	Con ^‡^	38.90 (11.81)	38.70 (11.20)			
Internal feeling	Int ^†^	11.08 (3.75)	12.27 (3.94)	Time Group Time × Group	0.014 * 0.355 0.031 *	0.019 0.003 0.015
	Con ^‡^	11.28 (4.21)	11.36 (4.44)			
Situational	Int ^†^	9.90 (3.75)	10.28 (3.71)	Time Group Time × Group	0.099 0.967 0.748	0.009 0.001 0.001
	Con ^‡^	9.79 (3.89)	10.35 (3.99)			
Competing demand	Int ^†^	9.64 (3.17)	10.30 (3.26)	Time Group Time × Group	0.108 0.227 0.240	0.008 0.005 0.004
	Con ^‡^	10.28 (3.72)	10.39 (3.53)			
Total METs	Int ^†^	61.55 (25.13)	64.09 (25.15)	Time Group Time × Group	0.252 0.107 0.565	0.004 0.008 0.001
	Con ^‡^	66.54 (27.27)	67.38 (27.20)			

* Significant difference (*p*-value < 0.05), ^†^ Intervention, ^‡^ Control.
